# Unraveling “Feeling Bad” in a Non-Western Culture: Achievement Emotions in Japanese Medical Students

**DOI:** 10.1007/s40670-025-02296-w

**Published:** 2025-01-29

**Authors:** Osamu Nomura, Momoka Sunohara, Haruko Akatsu, Jeffrey Wiseman, Susanne P. Lajoie

**Affiliations:** 1 Medical Education Development Center, Gifu University, Gifu, Japan; 2 Institute of Health Sciences Education, McGill University, Montreal, Canada; 3 Department of Political Science, Concordia University, Montreal, Canada; 4 International University of Health and Welfare, Tokyo, Japan; 5 Department of Educational & Counselling Psychology, McGill University, Montreal, Canada

**Keywords:** Achievement emotions, Culture, Control value theory, Clinical reasoning

## Abstract

**Introduction:**

The Medical Emotion Scale has been translated into Japanese (J-MES) and validated for cross-cultural emotion research in medical education. However, its applicability for extracting Japanese cultural aspects of medical students’ emotions has not been examined. This study aimed to explore the underlying latent constructs related to culture in the J-MES by conducting factor analyses.

**Methods:**

In total, 41 medical students enrolled at a Japanese university participated in this study. The students completed the J-MES before, during, and after a computer-based clinical reasoning activity. Exploratory factor analysis (EFA) was conducted to examine the factor structure of the scale. Factor extraction was based on a scree plot investigation.

**Results:**

The EFA for emotions before the task pointed to a four-factor structure explaining 56.70% of the total variance. The first factor accounted for 26.44% of the variance. Based on the seven items with the highest loadings on this factor (e.g., happiness), we interpreted the first factor as representing a positive valence dimension. The second factor explained 13.78% of the variance with four items of highest loadings (e.g., anger), which was interpreted as representing negative emotions toward the learning activity. The third factor explained 10.48% of the variance with three items (e.g., shame), interpreted as negative emotions related to self-performance. The fourth factor explained 6.00% of the variance with three items (e.g., confusion), which was interpreted as representing anxiety-related emotions.

**Discussion:**

Negative emotions included multiple factors such as learning activity- and self-performance-related emotions, which could be associated with Japan’s interdependent culture.

**Supplementary Information:**

The online version contains supplementary material available at 10.1007/s40670-025-02296-w.

## Introduction

Research examining the effects of physicians’ emotions on their performances and well-being is a burgeoning field [1]. The potential relevance of such research toward improving health-care practices, systems, and education is becoming more evident. Daily stress-related complaints stemming from physicians’ and medical students’ practice-related activities call for effective interventions. However, the emotions experienced in learning and achievement have rarely been discussed in medical education [2, 3], with existing studies focusing mostly on the impact of trainees’ negative emotions on mental health [4, 5]. Other research has focused on the effects of emotional training to enhance interpersonal skills in the workplace [6–8]. Research on the perceived stress of physicians has shown that negative emotions such as stress, anxiety, and fear can lead to poor clinical performance [9, 10].

Culture shapes how people experience and express emotions. Cultural psychology has illustrated that individuals’ way of perceiving and showing emotion is different across their own cultural contexts. [11, 12]. Historically, the majority of emotion research has been conducted in Western samples, often with White, educated samples, and the findings from these Western contexts were regarded as the “dominant norm” of individuals’ emotions [13, 14]. Contemporary scholars have reflected on the fact that the evidence from the limited samples is not generalizable to the global population [15]. Moreover, ignoring the cultural values of emotions may lead to negative consequences such as an accumulation of biased evidence and a lack of consideration for diversity in educational policy making. Therefore, emotion research in health sciences education should account for samples and perspectives from diverse cultural contexts; however, most published studies have been conducted in Western countries and only a few have been reported from Asian contexts. Furthermore, the development of instruments to measure emotions in medical contexts is still in its infancy and considerations for diverse cultures are needed.

### Theoretical Framework

One of the most comprehensive approaches known for interpreting emotions within the context of learning and academic achievement is Pekrun’s Control Value Theory [16, 17], which postulates that learners’ emotions influence the degree to which they successfully complete a given task. Learners’ emotions are regulated by their perceptions of the degrees to which they have control over and value for a given task, and their control and value perceptions are, in turn, influenced by factors in their learning environment.

The control-value theory categorizes emotions along several dimensions, including *valence*, *activation*, *object focus*, and *time frame*. Valence refers to whether emotions are pleasant (i.e., positive) or unpleasant (i.e., negative), such as enjoyment versus frustration. Activation signifies the degree of physiological arousal associated with emotions (i.e., activating or deactivating). Also, the objects that learners focus on, such as outcomes (e.g., performance) and learning environment (e.g., learning activity), are critical in the generation of achievement emotions. Furthermore, achievement emotions can arise from one’s focus on the time frame in which they occur, such as a prospective, concurrent, or retrospective.

Duffy et al. [18] developed and validated the Medical Emotion Scale (MES) based on control-value theory to measure a broad range of emotional states encountered in a Western medical education context. The MES is a self-report scale consisting of 20 items designed to measure different types of achievement emotions on a 5-point Likert scale to estimate the perceived intensity of each emotion. Each emotion is defined by valence (positive or negative) and arousal level (activating versus deactivating) to create four categories of medical practice-related emotions: (1) positive activating, (2) positive deactivating, (3) negative activating, and (4) negative deactivating. In addition, the MES is reported before, during, and after the assigned learning activity, based on the time frame aspect of control-value theory. Since its development, the MES has been used in numerous medical education studies to examine the impact of emotions on the performance of medical trainees in North America and Europe [19–22].

In medical education, only a few cross-cultural research studies of emotions in medical education have been conducted [23, 24]. The MES was previously translated into Japanese (J-MES) by the present authors to measure the emotions of Japanese medical trainees [25]. That validation study, which used Kane’s four-step framework (scoring, generalization, extrapolation, and implication) [26, 27], confirmed the linguistic equivalence between the Japanese and English versions and demonstrated evidence of validity for control-value theory to be well aligned with this scale. While the “scoring” and “generalization” steps of the J-MES validation were deeply evaluated, we concluded that further examinations of the “extrapolation” and “implication” steps of the J-MES validation would require collecting validity evidence regarding cultural differences between Eastern and Western countries, particularly in regard to the social emotion items of the J-MES (e.g., pride). Accordingly, further validation process of the J-MES is underway, to further examine these cultural considerations. Culture is a complicated concept, and it is challenging to provide a simple definition [29, 30]. No single psychometric measure can capture the complex and interacting elements of culture in medical educational contexts. However, a statistical factor analysis of the J-MES may help identify the “latent constructs” or “factors” underlying the scale that are related to culture based on the assumption that some emotions are sensitized by culture-dependent social interactions. In addition, because the expression of emotions in different cultures may be influenced by the time frames in which they occur [30, 31], examining how the cultural “latent constructs” extracted by factor analyses change before, during, and after a learning activity may reveal potential cultural effects.

Given this background, the present study aimed to explore the underlying latent constructs or factors related to culture in the J-MES by conducting three factor analyses (i.e., before, during, and after clinical reasoning activities) using the preexisting database from the initial J-MES validation study.

### Research Questions

To address the above research gap, we explored the following research question: “Are there any latent constructs, factors, or structures related to culture in the emotions of Japanese medical students perceived (a) before, (b) during, and (c) after a clinical reasoning activity as measured by the J-MES?”.

## Methods

### Design

The present study was a secondary analysis of a J-MES validation study composed of 20 items that was developed to measure different types of achievement emotions in medical education contexts using a 5-point Likert-type scale (from 1 = not at all to 5 = very strongly) [32, 33] (Supplement Fig. 1). The adjective items were categorized into four emotion groups according to their valence (positive/negative) and activation (activating/deactivating): (a) positively activating (e.g., happiness, enjoyment); (b) positively deactivating (e.g., relaxation, relief); (c) negatively activating (e.g., fear, shame), or (d) negatively deactivating (e.g., sadness, boredom). The descriptive statistics of the J-MES was shown in the Supplemental Table 1.

### Ethics and Consent

The study was reviewed and approved by the research ethics board at a North American university (No. 511–0518) and the research ethics committee at a Japanese medical school (No. 18-Im-005). Written informed consent was obtained from all participants.

### Participants and Learning Environment

The participants were selected from a cohort of second-year Japanese medical students (class size: 120 students) attending a medical school in Japan where the students receive preclinical medical education in English. At the time of recruitment, the students were informed that participation in the study was an entirely voluntary learning activity conducted outside regular class hours, as this activity was not part of the formal curriculum of the medical school. Forty-one students (*n* = 19 females) with a mean age of 22.3 (*SD* = 3.8) agreed to participate and completed the J-MES before, during, and after performing a computer-based clinical reasoning activity. All participants were learners enrolled in the preclinical curriculum taught in English. In this study, we utilized BioWorld [34], a computer-based learning software program that facilitates the development of medical students’ diagnostic reasoning skills through situating the diagnostic process in the context of solving medical cases.

### Analysis

We conducted three separate exploratory factor analyses (EFAs) to examine the factor structure of the J-MES using the observed emotion scores for each time point (before, during, and after a task). Promax rotation was adopted with the maximum likelihood method. Factor extraction was based on a scree plot investigation, and an item was accepted when the factor loading was ≥ 0.35. The Kaiser–Meyer–Olkin (KMO) measure of sampling and Bartlett’s test of sphericity were used to evaluate whether data were suitable for the EFAs. The KMO tests the adequacy of the sample size, and an average value > 0.6 is acceptable for the sample size in this study (i.e., < 100). Bartlett’s test of sphericity examines the suitability for the factor structure detection, and a significant value (< 0.05) is an indication that factor analysis is worthwhile for the database.

## Results

### Research Question 1a

**Fig. 1:**
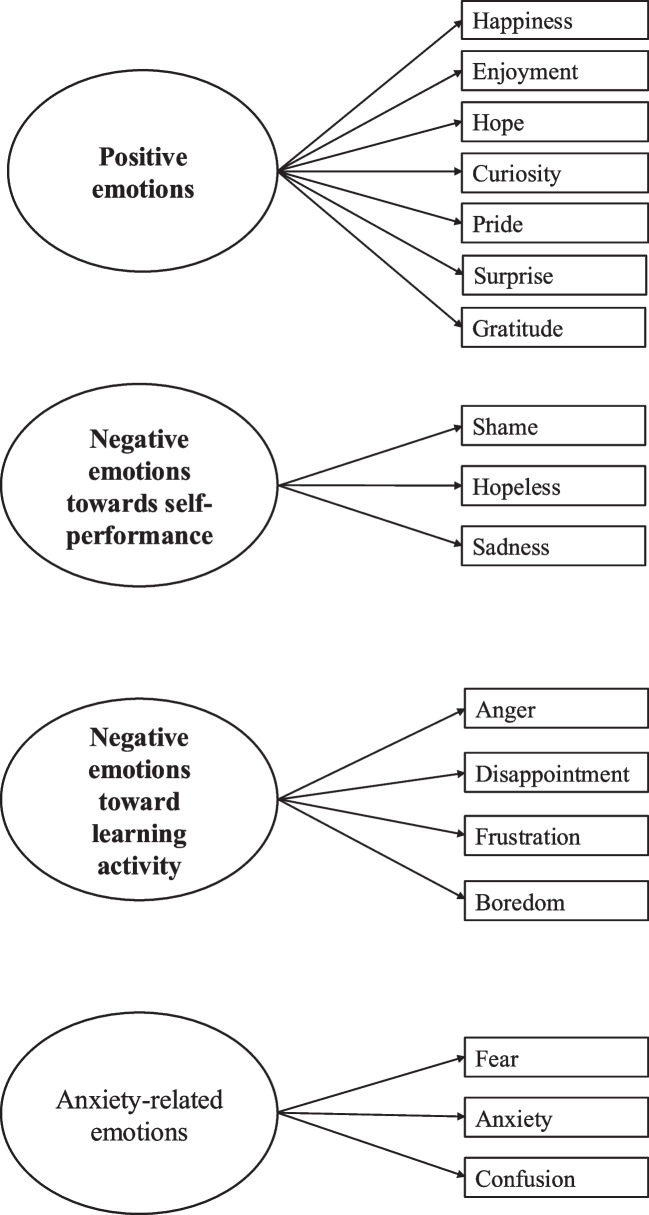
Factor analysis for emotions before after the clinical reasoning task

*Are there any latent constructs, factors, or structures related to culture in the emotions of Japanese medical students perceived before a clinical reasoning activity as measured by the J-MES?* (Fig. 1, Supplemental Table 2).

The EFA for emotions before the task revealed a KMO measure of sampling adequacy of 0.60. Bartlett’s test of sphericity was significant (*χ*^2^ (190) = 471.75, *p* < 0.001). The solution pointed to a four-factor structure explaining 56.70% of the total variance. After promax rotation, the first factor accounted for 26.44% of the variance. Based on the seven items with the highest loadings on this factor (happiness, enjoyment, hope, curiosity, pride, surprise, and gratitude), we interpreted the first factor as representing a positive valence dimension. The second factor explained 13.78% of the variance. Based on the four items with the highest loadings on this factor (anger, disappointment, frustration, and boredom), we interpreted the second factor as representing negative emotions toward the learning activity. The third factor explained 10.48% of the variance. Based on the items with the highest loadings on this factor (shame, hopeless, and sadness), we interpreted this factor as representing negative emotions related to self-performance. The fourth factor explained 6.00% of the variance. Based on the three items with the highest loadings on this factor (fear, anxiety, and confusion), we interpreted this factor as representing anxiety-related emotions. In sum, four factors including positive emotions, negative emotions toward the learning activity, negative emotions toward self-performance, and anxiety related emotions were extracted. These interpretations of emotion categories were defined based on the emotion dimensions (i.e., valence, activation, and object focus) of control-value theory.

### Research Question 1b

**Fig. 2:**
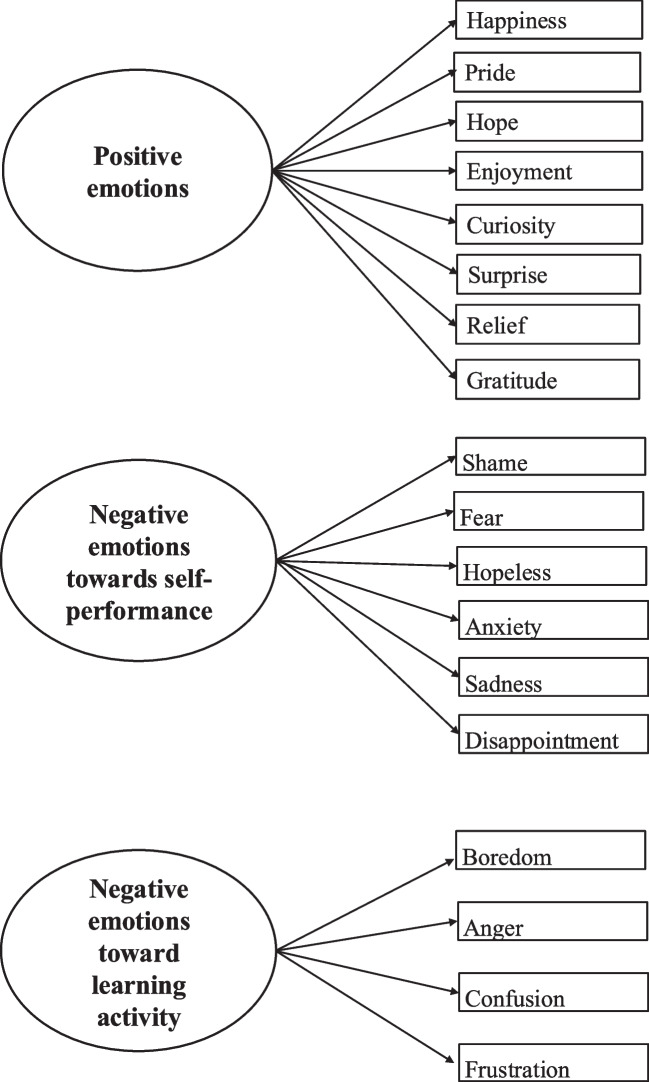
Factor analysis for emotions during the clinical reasoning task

*Are there any latent constructs, factors, or structures related to culture in the emotions of Japanese medical students perceived during a clinical reasoning activity as measured by the J-MES?* (Fig. 2, Supplemental Table 3).

In the second EFA for emotions experienced during the task, the KMO measure of sampling adequacy was 0.71 and Bartlett’s test of sphericity was significant (*χ*^2^ (190) = 508.49, *p* < 0.001). The solution pointed to a three-factor structure explaining 55.45% of the total variance. After promax rotation, the first factor accounted for 26.35% of the variance. Based on the six items with the highest loadings on this factor (shame, fear, hopeless, anxiety, sadness, and disappointment), we interpreted this factor as representing negative emotions related to self-performance. The second factor explained 20.35% of the variance. Based on the eight items with the highest loadings on this factor (happiness, pride, hope, enjoyment, curiosity, surprise, relief, and gratitude), we interpreted this factor as representing a positive valence dimension. The third factor explained 8.76% of the variance. Based on the four items with the highest loadings on this factor (boredom, anger, confusion, and frustration), we interpreted this factor as representing negative emotions toward the learning activity. In sum, four factors including negative emotions toward self-performance, positive emotions, and negative emotions toward the learning activity were extracted.

### Research Question 1c

**Fig. 3:**
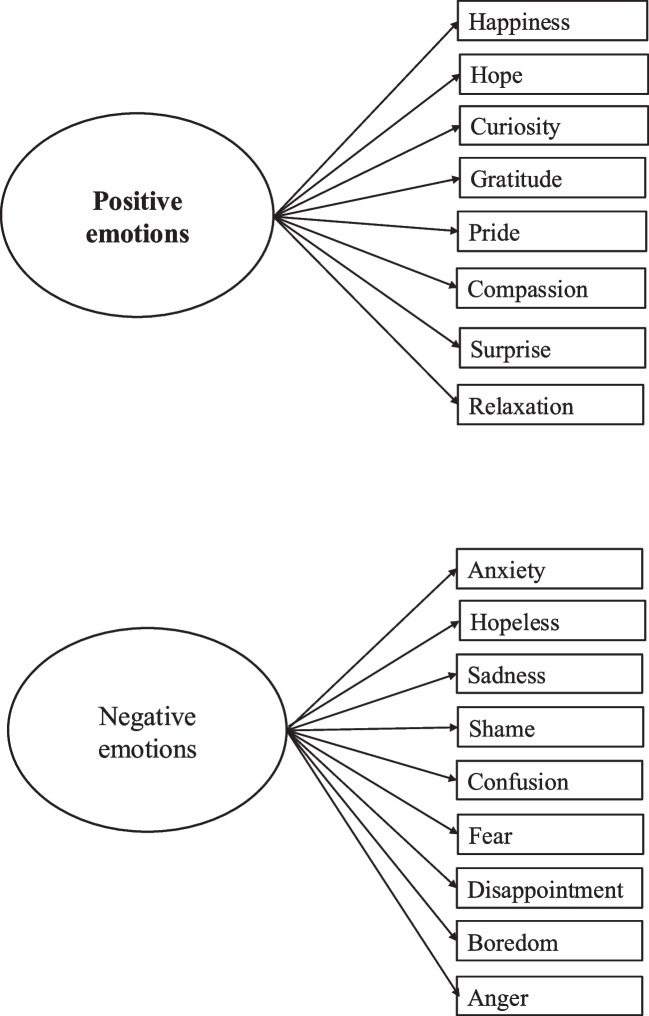
Factor analysis for emotions after the clinical reasoning task

*Are there any latent constructs, factors, or structures related to culture in the emotions of Japanese medical students perceived after a clinical reasoning activity as measured by the J-MES?* (Fig. 3, Supplemental Table 4).

In the third EFA of emotions after the task, the KMO measure of sampling adequacy was 0.76 and Bartlett’s test of sphericity was significant (*χ*^2^ (190) = 550.65, *p* < 0.001). The solution pointed to a two-factor structure that explained 51.82% of the total variance. After promax rotation, the first factor accounted for 32.02% of the variance. Based on the nine items with the highest loadings on this factor (anxiety, hopeless, sadness, shame, confusion, fear, disappointment, boredom, and anger), we interpreted the factor as representing a negative valence dimension. The second factor explained 19.81% of the variance. Based on the 10 items with the highest loadings on this factor (happiness, hope, curiosity, gratitude, pride, enjoyment, relief, compassion, surprise, and relaxation), we interpreted the factor as representing a positive valence dimension. In sum, two factors of negative and positive emotions were extracted.

## Discussion

This study explored the cultural latent constructs of the J-MES, and the results of the EFAs indicated that the component structures of emotions largely aligned with theoretical distinctions pertaining to positive and negative valence. Furthermore, as anticipated, the structures differed based on time frame (before, during, and after the clinical reasoning activity), which can be supported by the dimension of *focus* in the Control Value Theory [16, 17]. We also found that the negative emotions group was subdivided into two categories according to the object that learners focused on during the clinical reasoning training (i.e., self-performance and the learning activity). While the original MES study, which involved a Canadian sample, conducted a principal component analysis and found that emotions clearly aligned with a positive/negative dimension [18], it did not extract elements of objects that medical trainees focused on during the learning experiences, as in the present study.

The present study demonstrated that Japanese medical students distinguish their negative emotions directed toward their self-performance from those directed toward their learning activity or environment, indicating that another dimension in achievement emotions (i.e., object focus) was extracted from the data of Japanese medical students. This finding suggests a Japanese cultural value rooted in an interdependent cultural value as these findings have not been observed in the previous MES studies from Western contexts. In addition, the current findings may corroborate the empirical evidence from the field of cultural psychology proposing that East Asian cultures tend to prioritize an interdependent self, emphasizing harmony within the community over the personal autonomy and uniqueness [11, 12]. For example, Uchida et al. [35] examined cross-cultural differences in emotional agency between Japanese and American university contexts and reported that Japanese students considered emotions to be focused on relationships and occurring *among* individuals (the self and others/environment), whereas American students considered emotions to be focused on the self and occurring *within* individuals (i.e., the self only). Individual self-construal or self-concept is also reported to be linked to emotions and emotional regulation [30]. For instance, expressive suppression and cognitive reappraisal are common emotion regulation strategies [31]. Studies in the field of cultural psychology have suggested that people from Asian cultural contexts use expressive suppression more often than cognitive reappraisal [36, 37]. In addition, Japanese studies have reported that interpersonal (i.e., the self versus others) and environmental perceptions are important components of the expressive suppression of emotions [38, 39]. Through the accumulation of these findings, the cultural psychology literature suggests that people in East Asian cultural contexts tend to endorse an interdependent self, in which people view themselves as an extension of the larger group or community and prioritize maintaining a harmonious relationship with others and environments within their community over individual autonomy, uniqueness, and needs [36, 37]. Therefore, the present study provides new evidence regarding the validity of the cultural factors in the “extrapolation” step of Kane’s framework, as the cultural consideration was limited in our previous validation study of the J-MES.

The present results also suggest that the measurement tools in health professions that examine achievement emotions are, like any other educational measurement method, situated cultural artifacts [28, 29, 40]. This issue may be closely related to the final “implication” step of Kane’s validation framework. The MES, developed in a North American medical educational context, yields different results when applied to a Japanese medical educational context according to the current evidence that the dimension of object focus of achievement emotion was newly observed in the J-MES, which was not in the original English version of MES. Data from the J-MES revealed that Japanese medical students have a different “object focus” than do North American students when attempting to solve identical medical cases presented in identical software environments, suggesting a more interdependent culture that influences experienced emotions during clinical reasoning activities. Added dimensions of emotions postulated in control-value theory were identified in the present study, and the results indicate that the careful translation of measurements may create new value, such as the addition of a cultural construct.

This study has some limitations First, the current study’s small sample size, resulting from its process of voluntary recruitment for the informal learning activity, limited the generalizability of the results; thus, more participants could have led to a more statistical robustness of the findings. Secondly, the study institution was a Japanese medical school where courses are taught in English, which is an uncommon situation in Japan. Therefore, it is uncertain whether the emotional profile data in this study are representative of the general Japanese medical student population. Finally, we did not compare the J-MES data in Japanese with the MES data in Western contexts; thus, it was not possible to discuss cross-cultural comparisons. Further research is required with a larger sample size and participants from diverse cultural backgrounds to overcome the limitations of the present study.

In conclusion, when Japanese medical students “feel bad (i.e., negative emotions),” the object focus of the negative emotions may serve as a latent dimension in their achievement emotions. In other words, it is crucial for Japanese medical students whether the negative emotions are related to the assigned learning activity or to their performance. The present findings are supported not only by the Control Value Theory but also potentially associated with Japan’s interdependent culture. A larger sampled, cross-cultural comparison study is now needed to confirm the applicability and generalizability of the current insights.

## Supplementary Information

Below is the link to the electronic supplementary material.

Supplementary file1 (PDF 154 KB)

Supplementary file2 (PDF 78.7 KB)

## Data Availability

The data that support the findings of this study are available from the corresponding author upon reasonable request. The data are not publicly available due to privacy or ethical restrictions.

## References

[1] Choi W, Dyens O, Chan T, et al. Engagement and learning in simulation: recommendations of the Simnovate Engaged Learning Domain Group. BMJ Stel. 2017;3(Suppl 1):S23–32. 10.1136/bmjstel-2017-000232.

[2] McConnell MM, Eva KW. The role of emotion in the learning and transfer of clinical skills and knowledge. Acad Med. 2012;87:1316–22. 10.1097/ACM.0b013e318267644c. 22914515 10.1097/ACM.0b013e3182675af2

[3] O’Callaghan A. Emotional congruence in learning and health encounters in medicine: addressing an aspect of the hidden curriculum. Adv Health Sci Educ Theory Pract. 2013;18:305–17. 10.1007/s10459-012-9374-7. 22367055 10.1007/s10459-012-9353-4

[4] Dyrbye LN, Thomas MR, Shanafelt TD. Medical student distress: causes, consequences, and proposed solutions. Mayo Clin Proc. 2005;80:1613–22. 10.4065/80.12.1613. 16342655 10.4065/80.12.1613

[5] Shanafelt TD, Boone S, Tan L, et al. Burnout and satisfaction with work-life balance among US physicians relative to the general US population. Arch Intern Med. 2012;172:1377–85. 10.1001/archinternmed.2012.3199. 22911330 10.1001/archinternmed.2012.3199

[6] Arora S, Ashrafian H, Davis R, Athanasiou T, Darzi A, Sevdalis N. Emotional intelligence in medicine: a systematic review through the context of the ACGME competencies. Med Educ. 2010;44:749–64. 10.1111/j.1365-2923.2010.03609.x. 20633215 10.1111/j.1365-2923.2010.03709.x

[7] Satterfield JM, Hughes E. Emotion skills training for medical students: a systematic review. Med Educ. 2007;41:935–41. 10.1111/j.1365-2923.2007.02812.x. 17822414 10.1111/j.1365-2923.2007.02835.x

[8] Smith KB, Profetto-McGrath J, Cummings GG. Emotional intelligence and nursing: an integrative literature review. Int J Nurs Stud. 2009;46:1624–36. 10.1016/j.ijnurstu.2009.06.009. 19596323 10.1016/j.ijnurstu.2009.05.024

[9] LeBlanc VR. The effects of acute stress on performance: implications for health professions education. Acad Med. 2009;84:S25–33. 10.1097/ACM.0b013e3181b37b8f. 19907380 10.1097/ACM.0b013e3181b37b8f

[10] Harvey A, Bandiera G, Nathens AB, LeBlanc VR. Impact of stress on resident performance in simulated trauma scenarios. J Trauma Acute Care Surg. 2012;72:497–503. 10.1097/TA.0b013e3182415efc. 22439221 10.1097/ta.0b013e31821f84be

[11] Markus HR, Kitayama S. Culture and the self: implications for cognition, emotion, and motivation. Psychol Rev. 1991;98:224–53. 10.1037/0033-295X.98.2.224.

[12] Markus HR, Kitayama S. Cultures and selves: a cycle of mutual constitution. Perspect Psychol Sci. 2010;5:420–30. 10.1177/1745691610375557. 26162188 10.1177/1745691610375557

[13] Spinrad, T. L., & Eisenberg, N. (2024). The socialization of emotion regulation. In J. J. Gross & B. Q. Ford (Eds.), *Handbook of emotion regulation* (3rd ed., pp. 129–135). The Guilford Press.

[14] Chentsova Dutton, Y., Tuna, E., & Tamir, M. (2024). Emotion regulation and psychopathology across cultures. In J. J. Gross & B. Q. Ford (Eds.), *Handbook of emotion regulation* (3rd ed., pp. 322–328). The Guilford Press.

[15] Seligman, R. (2024). Anthropology and Emotion Regulation. In J. J. Gross & B. Q. Ford (Eds.), *Handbook of emotion regulation* (3rd ed., pp. 552–558). The Guilford Press.

[16] Pekrun R. The control-value theory of achievement emotions: assumptions, corollaries, and implications for educational research and practice. Educ Psychol Rev. 2006;18:315–41. 10.1007/s10648-006-9029-9.

[17] Pekrun R. Control-value theory: from achievement emotion to a general theory of human emotions. Educ Psychol Rev. 2024;36(3):83.

[18] Duffy MC, Lajoie SP, Pekrun R, Lachapelle K. Emotions in medical education: examining the validity of the Medical Emotion Scale (MES) across authentic medical learning environments. Learn Instr. 2020;70: 101150. 10.1016/j.learninstruc.2020.101150.

[19] Lajoie SP, Zheng J, Li S. Examining the role of self-regulation and emotion in clinical reasoning: implications for developing expertise. Med Teach. 2018;40:842–4. 10.1080/0142159X.2018.1479138. 29947294 10.1080/0142159X.2018.1484084

[20] Harley JM, Jarrell A, Lajoie SP. Emotion regulation tendencies, achievement emotions, and physiological arousal in a medical diagnostic reasoning simulation. Instr Sci. 2019;47:151–80. 10.1007/s11251-019-09515-8.

[21] Lajoie SP, Zheng J, Li S, Jarrell A, Gube M. Examining the interplay of affect and self-regulation in the context of clinical reasoning. Learn Instr. 2019;72: 101219. 10.1016/j.learninstruc.2019.101219.

[22] Polujanski S, Schindler AK, Rotthoff T. Academic-associated emotions before and during the COVID-19-related online semester: a longitudinal investigation of first-year medical students. GMS J Med Educ. 2020;37:Doc77. 10.3205/zma001396 10.3205/zma001370 PMC7740038 33364356

[23] Helmich E, Yeh HM, Kalet A, Al-Eraky M. Becoming a doctor in different cultures: toward a cross-cultural approach to supporting professional identity formation in medicine. Acad Med. 2017;92:58–62. 10.1097/ACM.0000000000001396. 27782917 10.1097/ACM.0000000000001432

[24] Helmich E, Yeh HM, Yeh CC, de Vries J, Tsai DFC, Dornan T. Emotional learning and identity development in medicine: a cross-cultural qualitative study comparing Taiwanese and Dutch medical undergraduates. Acad Med. 2017;92:853–9. 10.1097/ACM.0000000000001541. 28353499 10.1097/ACM.0000000000001658

[25] Nomura O, Wiseman J, Sunohara M, Akatsu H, Lajoie SP. Japanese medical learners’ achievement emotions: accounting for culture in translating Western medical educational theories and instruments into an Asian context. Adv Health Sci Educ Theory Pract. 2021;26:1255–76. 10.1007/s10459-021-10014-0. 33978878 10.1007/s10459-021-10048-9 PMC8452569

[26] Cook DA, Brydges R, Ginsburg S, Hatala R. A contemporary approach to validity arguments: a practical guide to K ane’s framework. Med Educ. 2015;49(6):560–75. 25989405 10.1111/medu.12678

[27] Cordovani, L., Jack, S. M., Wong, A., & Monteiro, S. (2024). Surveying undergraduate medical students’ motivational orientations and learning strategies in the first and last year of medical school. *Medical Science Educator*, 1–11. 10.1007/s40670-024-02067-z PMC11297228 39099868

[28] Watling CJ, Ajjawi R, Bearman M. Approaching culture in medical education: three perspectives. Med Educ. 2020;54:289–95. 10.1111/medu.14082. 31872497 10.1111/medu.14037

[29] Meyer, E. (2014). *The culture map: Breaking through the invisible boundaries of global business*. Public Affairs.

[30] Ford BQ, Mauss IB. Culture and emotion regulation. Curr Opin Psychol. 2015;1:1–5. 10.1016/j.copsyc.2015.03.016. 10.1016/j.copsyc.2014.12.004 PMC4341898 25729757

[31] Gross JJ, John OP. Individual differences in two emotion regulation processes: implications for affect, relationships, and well-being. J Pers Soc Psychol. 2003;85(2):348–62. 10.1037/0022-3514.85.2.348. 12916575 10.1037/0022-3514.85.2.348

[32] Nomura O, Soma Y, Ikezaki Y, Tazoe H, Osanai M, Hosokawa S, Tomisawa T. Effect of virtual-reality-based training on emotions of medical students undertaking radiation emergency medicine: an educational experimental study. Disaster Med Public Health Prep. 2024;18: e198. 39463289 10.1017/dmp.2024.166

[33] Nomura O, Aoyagi A, Irie J, Goto T, Sugiyama K, Hanada H, Ishizawa Y. Positive emotions for promoting quality improvement of extracorporeal membrane oxygenation therapy for COVID-19: in situ interprofessional simulation. Acute Medicine & Surgery. 2024;11(1): e70002. 39185273 10.1002/ams2.70002 PMC11342042

[34] Lajoie SP. Student modeling for individuals and groups: the BioWorld and HOWARD platforms. Int J Artif Intell Educ. 2021;31:460–75. 10.1007/s40593-020-00219-x.

[35] Uchida Y, Kitayama S. Happiness and unhappiness in east and west: themes and variations. Emotion. 2009;9:441–56. 10.1037/a0015634. 19653765 10.1037/a0015634

[36] Matsumoto D. Are cultural differences in emotion regulation mediated by personality traits? J Cross-Cult Psychol. 2006;37:421–37. 10.1177/0022022106288471.

[37] Matsumoto D, Yoo SH, Nakagawa S, 37 members of the Multinational Study of Cultural Display Rules. Culture, emotion regulation, and adjustment. J Pers Soc Psychol. 2008;94:925–37. 10.1037/0022-3514.94.6.925 10.1037/0022-3514.94.6.925 18505309

[38] Imada T, Ellsworth PC. Proud Americans and lucky Japanese: cultural differences in appraisal and corresponding emotion. Emotion. 2011;11:329–45. 10.1037/a0022505. 21500902 10.1037/a0022855

[39] Nakamura M. Display and decoding rules in the communication of emotion: a conceptual analysis and cross-cultural questionnaire study. Bull Fac Hum Sci Osaka Univ. 1991;17:115–45.

[40] Cantillon P, De Grave W, Dornan T. The social construction of teacher and learner identities in medicine and surgery. Med Educ. 2022;56:614–24. 10.1111/medu.14528. 34993973 10.1111/medu.14727 PMC9305233

