# Supplementary material for: Unraveling “Feeling Bad” in a Non-Western Culture: Achievement Emotions in Japanese Medical Students
Source: Med Sci Educ. 2025 Jan 29;35(3):1259–67. doi: 10.1007/s40670-025-02296-w (PMC12228877; doi:10.1007/s40670-025-02296-w)
Supplement: Supplementary file 1 — Supplementary file1 (PDF 154 KB) [file 40670_2025_2296_MOESM1_ESM.pdf]

	1<br>Not at all	2<br>Very little	3<br>Moderate	4<br>Strong	5<br>Very strong
Confused	○	○	○	○	○
Hopeful	○	○	○	○	○
Bored	○	○	○	○	○
Proud	○	○	○	○	○
Sad	○	○	○	○	○
Anxious	○	○	○	○	○
Happy	○	○	○	○	○
Frustrated	○	○	○	○	○
Hopeless	○	○	○	○	○
Enjoyment	○	○	○	○	○
Ashamed	○	○	○	○	○
Compassionate	○	○	○	○	○
Surprised	○	○	○	○	○
Curious	○	○	○	○	○
Afraid	○	○	○	○	○
Grateful	○	○	○	○	○
Disappointed	○	○	○	○	○
Relieved	○	○	○	○	○
Angry	○	○	○	○	○
Relax	○	○	○	○	○

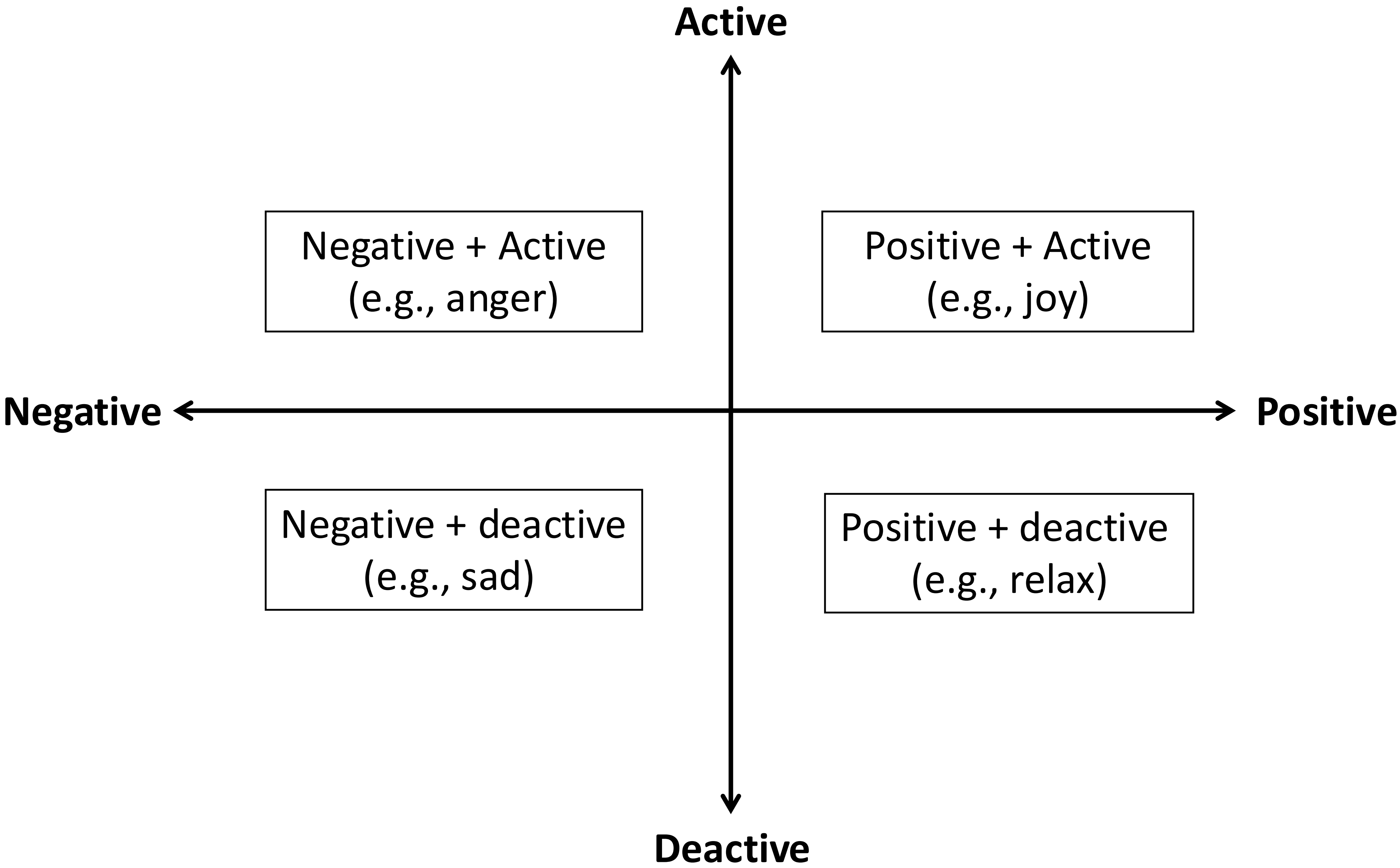
Active
Negative + Active
(e.g., anger)
Positive + Active
(e.g., joy)
Negative
Positive
Negative + deactive
(e.g., sad)
Positive + deactive
(e.g., relax)
Deactive
