# Supplementary material for: Unraveling “Feeling Bad” in a Non-Western Culture: Achievement Emotions in Japanese Medical Students
Source: Med Sci Educ. 2025 Jan 29;35(3):1259–67. doi: 10.1007/s40670-025-02296-w (PMC12228877; doi:10.1007/s40670-025-02296-w)
Supplement: Supplementary file 2 — Supplementary file2 (PDF 78.7 KB) [file 40670_2025_2296_MOESM2_ESM.pdf]

Supplement Table 1.

Descriptive statistics of emotions before, during and after the task

	Before	During	After
Item	*M* (*SD*)	*M* (*SD*)	*M* (*SD*)
Happiness	2.8 (1.3)	2.6 (1.3)	2.5 (1.3)
Enjoyment	3.1 (1.1)	3.7 (1.1)	3.1 (1.3)
Hope	3.4 (1.1)	3.0 (1.1)	3.0 (1.2)
Curiosity	3.8 (1.1)	3.6 (1.1)	3.2 (1.3)
Pride	2.5 (1.3)	2.2 (1.3)	2.5 (1.4)
Surprise	2.4 (1.4)	2.4 (1.2)	2.2 (1.2)
Gratitude	2.5 (1.3)	2.1 (1.2)	2.7 (1.4)
Anger	1.2 (0.5)	1.3 (0.6)	1.3 (0.6)
Disappointment	1.3 (0.6)	1.6 (1.0)	1.7 (1.0)
Frustration	1.5 (0.8)	1.6 (0.9)	1.5 (0.9)
Boredom	1.5 (0.7)	1.7 (0.9)	1.6 (0.9)
Shame	1.6 (1.0)	2.0 (1.1)	1.9 (1.2)
Hopeless	1.8 (1.1)	2.1 (1.0)	1.8 (1.1)

Sadness	1.2 (0.5)	1.9 (1.1)	1.6 (1.1)
Fear	1.7 (0.9)	1.8 (0.9)	1.5 (1.0)
Anxiety	2.3 (1.1)	2.7 (1.2)	2.1 (1.3)
Confusion	2.1 (1.1)	2.5 (1.2)	1.9 (1.2)
Compassion	1.9 (1.0)	2.0 (1.0)	1.9 (1.1)
Relief	2.5 (1.2)	2.5 (1.2)	3.0 (1.3)
Relaxation	3.4 (1.0)	3.4 (1.1)	3.4 (1.3)

Supplemental table 2. Summary of the exploratory factor analysis results for emotions *before* the task

	Factor loadings			
Item	Positive emotions	Negative emotions toward learning activity	Negative emotions towards self-performance	Anxiety-related emotions
Happiness	**.90**	.12	–.08	.07
Enjoyment	**.82**	–.06	.22	–.19
Hope	**.78**	.02	–.31	.24
Curiosity	**.75**	–.32	.23	–.12
Pride	**.69**	.09	–.22	.01
Surprise	**.61**	.04	.22	.11
Gratitude	**.56**	–.02	–.26	–.26
Anger	.12	**.88**	.03	–.03
Disappointment	–.02	**.83**	.02	–.17
Frustration	.01	**.79**	–.12	–.01
Boredom	–.09	**.69**	.01	.11
Shame	.05	–.11	**.76**	–.17

Hopeless	–.13	–.02	**.74**	.04
Sadness	.18	.47	**.64**	.05
Fear	.44	–.05	.01	**.66**
Anxiety	.48	–.07	.05	**.58**
Confusion	–.10	.15	.33	**.39**
Compassion	–.08	.14	–.30	–.10
Relief	.31	.23	–.16	–.49
Relaxation	.22	–.08	.18	–.51
Eigenvalues	5.69	3.09	2.62	1.63
% of variance	26.44	13.78	10.48	6.00

Factor loadings > 0.35 appear in bold.

Supplemental table 3. Summary of the exploratory factor analysis results for emotions *during* the task

	Factor loadings

Item	Negative emotions towards self-performance	Positive emotions	Negative emotions toward learning activity
Shame	**.97**	–.40	–.23
Fear	**.88**	.16	–.23
Hopeless	**.76**	.02	.17
Anxiety	**.67**	.40	.17
Sadness	**.65**	–.06	.26
Disappointment	**.48**	–.08	.38
Happiness	–.11	**.95**	.08
Pride	.10	**.79**	.27
Hope	–.01	**.78**	.00
Enjoyment	–.31	**.68**	–.03
Curiosity	–.10	**.66**	–.26
Surprise	.50	**.63**	–.06
Relief	–.45	**.43**	.18
Gratitude	.18	**.42**	–.15

Boredom	–.25	–.02	**.99**
Anger	–.03	.05	**.61**
Confusion	.28	–.13	**.58**
Frustration	.02	.12	**.52**
Compassion	.06	.28	.01
Relaxation	–.40	.29	–.14
Eigenvalues	5.91	4.24	2.13
% of variance	26.35	20.35	8.76

Factor loadings > 0.35 appear in bold.

Supplemental table 4. Summary of the exploratory factor analysis results for emotions *after* the task

	Factor loadings	
Item	Negative emotions	Positive emotions

Anxiety	**.91**	–.05
Hopeless	**.87**	–.21
Sadness	**.85**	–.15
Shame	**.81**	–.06
Confusion	**.76**	–.37
Fear	**.65**	–.10
Disappointment	**.63**	–.29
Boredom	**.54**	–.43
Anger	**.39**	–.14
Happiness	–.30	**.88**
Hope	–.21	**.86**
Curiosity	–.19	**.82**
Gratitude	–.23	**.77**
Pride	–.13	**.69**
Enjoyment	–.10	**.64**
Relief	–.49	**.62**
Compassion	–.20	**.53**

Surprise	.12	**.52**
Relaxation	–.56	**.42**
Frustration	.22	–.42
Eigenvalues	7.12	4.12
% of variance	32.02	19.81

Factor loadings > 0.35 appear in bold.
